# Neural serotonergic circuits for controlling long-term voluntary alcohol consumption in mice

**DOI:** 10.1038/s41380-022-01789-z

**Published:** 2022-10-04

**Authors:** Arnauld Belmer, Ronan Depoortere, Kate Beecher, Adrian Newman-Tancredi, Selena E. Bartlett

**Affiliations:** 1 Addiction and Obesity Laboratory, Translational Research Institute, Faculty of Health, School of Clinical Sciences, Queensland University of Technology, Brisbane, QLD Australia; 2 Neurolixis SAS, 81100 Castres, France

**Keywords:** Neuroscience, Physiology

## Abstract

Alcohol-use-disorders are chronic relapsing illnesses, often co-morbid with anxiety. We have previously shown using the “drinking-in-the-dark” model in mice that the stimulation of the serotonin receptor 1A (5-HT_1A_) reduces ethanol binge-drinking behaviour and withdrawal-induced anxiety. The 5-HT_1A_ receptor is located either on Raphe neurons as autoreceptors, or on target neurons as heteroreceptors. By combining a pharmacological approach with biased agonists targeting the 5-HT_1A_ auto- or heteroreceptor and a chemogenetic approach (DREADDs), here we identified that ethanol-binge drinking behaviour is dependent on 5-HT_1A_ autoreceptors and 5-HT neuronal function, with a transition from DRN-dependent regulation of short-term (6 weeks) ethanol intake, to MRN-dependent regulation after longer ethanol exposure (12 weeks). We further identified a serotonergic microcircuit (5-HT^MRN→DG^) originating from the MRN and projecting to the dentate gyrus (DG) of the hippocampus, that is specifically affected by, and modulates long-term ethanol consumption. The present study indicates that targeting Raphe nuclei 5-HT_1A_ autoreceptors with agonists might represent an innovative pharmacotherapeutic strategy to combat alcohol abuse.

## Main

Alcohol-use-disorders (AUDs) are chronic relapsing illnesses, with alcohol often self-medicated to cope with the distressing symptoms of various mental health issues. The COVID-19 pandemic led to greater reports of stress- and fear-related mental health issues, possibly contributing to increases in worldwide alcohol consumption [1–5]. Given that the brain serotonin (5-Hydroxytryptamine, 5-HT) system is closely involved in the stress responses to social isolation [6, 7], fear [8, 9], anxiety [9, 10] and depression [11], we hypothesized that alterations in brain 5-HT neurotransmission contributes to the reinforcement of alcohol seeking/drinking behaviour. Research studies have shown that manipulation of the neural activity of 5-HT neurons from the dorsal or median Raphe nuclei (DRN or MRN, respectively) alters anxiety-like behaviour, antidepressant-like and anti-impulsive-like effects [12–15], arousal states [16, 17], sleep cycles [18, 19], response to rewarding or aversive stimuli [20–23], social behaviour and aggression [16, 24]. Since most of these behaviours are also affected by alcohol consumption, it is likely that 5-HT neuroplasticity plays a role in the maintenance of long-term alcohol consumption [25].

Previous studies from our laboratory and others have revealed that long-term alcohol consumption alters the expression and function of serotonin 1A (5-HT_1A_) autoreceptors (i.e., those located on Raphe nuclei) and heteroreceptors (i.e., those located on projection brain regions) [26–28], as well as the morphology [29] and function [30] of Raphe nuclei 5-HT neurons. We further demonstrated that a chronic treatment with pindolol or tandospirone, partial and more efficacious 5-HT_1A_ receptor agonists, respectively, reduces ethanol intake, prevents withdrawal-induced anxiety-like behaviour and reverses the deficits in hippocampal neurogenesis elicited by long-term ethanol intake in mice [28, 31]. However, the specific contribution of 5-HT_1A_ auto- vs heteroreceptors, and the role played by Raphe nuclei 5-HT neurotransmission in alcohol drinking behaviour is less well understood. In the present study, we used NLX-112 (a.k.a. befiradol or F13640), a highly selective unbiased 5-HT_1A_ agonist (i.e., with no marked preference for auto- vs heteroreceptors), or biased agonists that preferentially target 5-HT_1A_ autoreceptors (F13714) or heteroreceptors (NLX-101, a.k.a. F5599) [32–34] to determine whether 5-HT_1A_ auto- or heteroreceptors mediate the effect of 5-HT_1A_ receptor agonists on the reduction of ethanol intake. The molecular basis for the auto/heteroreceptor selectivity of these 5-HT_1A_ receptor biased agonists appears to be related to preferential activation of specific G-protein subtypes in different brain regions [35–37]. Evidence supporting the differential coupling to G-proteins and consequent differential activation of downstream signalling cascades has been reviewed extensively [38]. We identified 5-HT_1A_ autoreceptors as the receptor subpopulation mediating both short- and long-term ethanol intake. Brain cannulation and local microinjections of these agonists allowed us to identify which nucleus, DRN or MRN, mediates the 5-HT_1A_ receptor-dependent reduction in ethanol intake following short- vs long-term exposure in the “drinking in the dark” model in mice. We further used chemogenetic manipulation of *pet1*-5-HT neuron activity with CRE-dependent Designer Receptors Exclusively Activated by Designer Drugs (DREADDs), in combination with systemic or local injections of the designer drug Clozapine-N-Oxide (CNO) to identify the serotonergic circuits that control ethanol intake following long-term exposure. Lastly, we also assessed the systemic effects of NLX-112 and F13714, and intracranial effects (Raphe nuclei microinjection) of the latter on food (chow) and water intake, as well as on locomotor activity in naïve mice, to control for unspecific effects.

Using this double pharmacological and chemogenetic strategy, we discovered that the 5-HT neuronal circuits involved in ethanol consumption switched after long-term exposure, from DRN to MRN, and suggests that selective 5-HT_1A_ receptor biased agonists targeting those particular circuits might represent viable pharmacotherapeutics for the treatment of AUDs.

## Methods

### Animals and housing

Five-week-old male C57BL/6 J mice (ARC, WA, Australia) or hemizygous *pet1*-CRE mice [39] (Strain number: 012712, B6.Cg-Tg(Fev-cre)1Esd/J, JaxMice, USA) were individually housed under reverse light cycle conditions (lights off from 9:00 am to 9:00 pm) in a climate-controlled room (22 °C, 50 % humidity) with ad libitum access to food and filtered tap water. For each experiment involving animals, sample size was chosen according to previous studies from the laboratory. Following 1 week of habituation to the housing conditions, mice were offered alcohol during drinking-in-the-dark sessions. The *Pet1*-CRE+/0 allele was detected by PCR with the following primers: 5ʹ- CTTCTGTCCGTTTGCCGGTCGTGG / TTTTGCACGTTCACC GGCATCAACG -3ʹ that amplified a band of 264 bp as previously described [40]. All experimental procedures were approved by The University of Queensland and The Queensland University of Technology Animal Ethics Committees and complied with the policies and regulations regarding animal experimentation and other ethical matters, in accordance with the Queensland Government Animal Research Act 2001, associated Animal Care and Protection Regulations (2002 and 2008), as well as the Australian Code for the Care and Use of Animals for Scientific Purposes, 8th Edition (National Health and Medical Research Council, 2013).

### “Drinking-in-the-dark” ethanol intake model

We adapted the “Drinking-In-the-Dark” (DID) model of binge-like alcohol [41] or sucrose consumption with long-term exposure as previously described [27, 28, 31]. Mice were given access to one bottle of 20% (v/v) alcohol for a 2 h period (12 pm to 2 pm), 3 h into the dark cycle, Monday to Friday. Filtered water was available at all other times. The alcohol solution was presented in 50 ml plastic falcon tubes (Corning Centristar, NY, USA) fitted with rubber stoppers and a 6.35 cm stainless-steel sipper tube with double ball bearings. Alcohol containing tubes were weighed prior to and 2 h after presentation. For DREADD/DG-CNO injection, alcohol containing tube were weighed prior to, and 30 min and 2 h after presentation. Mouse weights were measured daily for 12 weeks to calculate the adjusted g/kg intake.

### Drugs

Drug administration occurred at 6 and 12 weeks in the same groups of animals. NLX-112 (befiradol or F13640; 3-chloro-4-fluorophenyl- [4-fluoro-4-([(5-methylpyridin-2-yl)methylamino]methyl) piperidin-1-yl]methanone, fumarate salt), NLX-101 (F15599; (3-Chloro-4-fluorophenyl-(4-fluoro-4-{[(5-methylpyrimidin-2-ylmethyl)-amino]-methyl}-piperidin-1-yl)-methanone, fumarate salt)) and F13714 (3-chloro-4-fluorophenyl-(4-fluoro-4-{[(5-methyl-6-methylaminopyridin-2-ylmethyl)-amino]-methyl}-piperidin-1-yl-methanone, fumarate salt)) and WAY-100,635 maleate (N-[2-[4-(2-methoxyphenyl)-1-piperazinyl]ethyl]- N-(2-pyridyl) cyclohexanecarboxamide, maleate salt). NLX-101, F13714 and NLX-112 were provided by Neurolixis; WAY-100,635 was commercially obtained from Abcam (ab120550). NLX compounds and WAY-100,635 were dissolved in 0.9% (w/v) sterile sodium chloride to 0.64 mg/kg; NLX drugs were then serially diluted to 0.04 mg/kg. Clozapine-N-oxide was purchased from AK scientific (Melbourne, Australia) and dissolved in 2% dimethyl sulfoxide (DMSO) in 0.9% (w/v) sterile sodium chloride. Tandospirone hydrochloride was purchased from Tocris (Cat. No. 2854, Tocris, Australia), dissolved in 2% dimethyl sulfoxide (DMSO) in 0.9% (w/v) sterile sodium chloride and chronically injected intraperitoneally (3 mg/kg/day). For brain infusion experiments, NLX compounds (1, 16 and 32 μg) and CNO (10 and 100 μM) were dissolved in artificial cerebro-spinal fluid, aCSF (in mM: 130 NaCl, 3 KCl, 26 NaHCO3, 1.25 NaH2PO4, 5 MgCl2, 10 D-glucose). All doses refer to the weight of the free base. All the drugs were tested by an experimenter blind to the treatment on alcohol consumption by intraperitoneal (i.p.) injections (10 ml/kg, 30 min prior to presentation of the bottles) or local brain infusion (0.1 μl/min, 15 min prior presentation of the bottles) in a pseudo random Latin-square design, where each mouse received each of the three doses of the drug over the testings, with each mouse serving as its own control. Systemic active doses of NLX-101, F13714, NLX-112 and WAY-100,635 were selected based on previous studies in rodents [38, 42, 43]. Intracranial doses of F13714 were based on previous publications [38, 43, 44].

### Locomotor activity

Ethanol-naïve 10-week-old mice were habituated 90 min/day, for 3 consecutive days, in the locomotor activity recording box (35 L × 35 W × 50 H cm^3^) and their activity video-tracked using the Any-Maze software. Day 1 was habituation to the box, Day 2 and 3 were habituation to the box with i.p. of saline solution 30 min into the start of the 90 min session. On Day 4, mice were treated with 0.64 mg/kg i.p. of the 5-HT_1A_ receptor agonists (NLX-101, NLX-112 or F13714) or saline, and locomotor activity was recorded for 150 min post-injection. The effect of local CNO (100 μM) microinjection in the hippocampus on locomotor activity was tested similarly in long-term ethanol-exposed MR-DREADDed mice in a subgroup of *n* = 4 mice, previously tested for ethanol intake, following one week of drug-washout period.

### Stereotaxic Surgeries

Following 4 weeks (short-term) or 10 weeks (long-term) of “drinking-in-the-dark” alcohol intake, mice underwent stereotaxic surgeries with implantation of guide cannula, microinjection of AAV-DREADD or both procedures. Guide cannula: Mice under isoflurane anaesthesia (2-5%, 1 L/min oxygen) were unilaterally (right) implanted with a guide cannula (26 gauge, PlasticOne) sitting 0.5 mm above the DRN (AP -4.5 mm, ML + 1.4 mm, DV 2.8 mm from bregma with a 25° medio-lateral angle [45]) or the MRN (AP -4.5 mm, ML + 1.25 mm, DV 4.0 mm from bregma, with a 15° medio-lateral angle), or bilaterally in the dentate gyri of the hippocampus (AP -2.0 mm, ML ± 1.5 mm, DV 1.5 mm from bregma). During 2 weeks of recovery, handling, and procedure habituation, mice continued the ethanol drinking procedure. **AAV-DREADDs** microinjection**:** CRE-dependent excitatory (AAV9-DIO-hSyn-**hM3Dq**-mCherry), inhibitory (AAV9-DIO-hSyn-**hM4Di**-mCherry) or control (AAV9-DIO-hSyn-mCherry) DREADDs constructs were purchased from Addgene (#50459, #44361, and #44362, titers ≥1 × 10¹³ vg/mL, USA). AAV vectors were microinjected in the DRN or MRN at the aforementioned coordinates. Three injections of 0.33 μl each were done along 3 different depths (1 μl total) along the dorso-ventral axis (+0.15, 0 and -0.15 mm from targeted region) at 10 nl/s using a Nanoject III (Drummond Scientific, Adelab). Three weeks after AAV-DREADD infection, CNO was administered i.p. at 1 mg/kg. Three weeks after cannulation with or without viral infection, 0.5 ul of F13714 or CNO was administered by a microinjection cannula (33 gauge) protruding 0.5 mm beyond the tip of the guide cannula, at a flow rate of 0.2 ul/min, in their home cage, with the injection cannula left in place for 5 min prior to removal. Ethanol bottles were presented in the DID 15 min after drug infusion.

### Histology

Mice were transcardially perfused with a 4% (w/v) paraformaldehyde solution, their brains extracted and post-fixed overnight at 4 °C. Brains were sectioned on a vibratome (VT1200S, Leica Biosystem, Australia), 40 μm free-floating coronal sections were used for microinjection cannula placement verification by light microscopy, or for immunohistochemistry experiments. Only animals with cannula correctly placed or viral vector correctly expressed in the target brain region were included in the analysis.

### Immunohistochemistry

Sections were incubated in permeabilization solution (Phosphate-buffer-saline 0.1 M, PBS; 1% TritonX-100; 0.1% Tween-20) for 1 h at room temperature, and rinsed in 2 × 5 min washes in antigen-retrieval (AR) solution (10 mM sodium citrate, 0.05%, tween-20 pH = 6.0), placed in a prewarmed AR solution at 80 °C for 30 min, cooled down to room temperature, and transferred to blocking solution (0.1 M PBS, 0.3 % Triton-X100, 0.05% Tween-20, 2% Normal Goat Serum) for 1 h at room temperature. For DREADDs infection site verification, the following primary antibodies were incubated 48 h at room temperature: mCherry (guinea pig anti-RFP 1:1000, Synaptic System #390 004), tryptophane hydroxylase 2 (Mouse anti-TPH2 antibody, Merck MAB91108, 1:500). After 3 × 5 min washes in blocking solution, corresponding secondary antibodies were incubated in blocking solution for 4 h at room temperature (goat anti-guinea pig–Alexa 594, Thermofisher # A-11076, 1:500; Goat anti-mouse biotinylated, Jackson Immunoresearch #115-065-166, 1:200). Sections were rinsed in PBST (0.1 M PBS, 0.3% Triton-X100, 0.03% Tween-20), and incubated in Streptavidin-Alexa 488 (Thermofisher, S11223, 1:1000) diluted in PBST, at room temperature for 30 min, rinsed 3 × 5 min in PBST, incubated in DAPI diluted in PBS (Thermofisher, #D1306, 1:1000), rinsed 3 × 5 min in PBS and mounted on slide with Prolong Gold antifade (Thermofisher, #P10144). For 5-HT innervation, immunohistochemistry was performed by a blind experimenter as previously described [46, 47]. Briefly, after permeabilization and blocking as above, sections were incubated with rat anti-5-HT antibody (Merck Millipore MAB352, 1:100) for 72 h at room temperature, followed by goat anti-rat biotinylated secondary antibody (Jackson Immunoresearch # 112-065-003, 1:200) for 4 h, and 30 min incubation with streptavidin-Cy3 (Thermofisher, #434315, 1:1000).

### Statistical analyses

Data are expressed as the mean with SEM or individual values (DREADD experiments). GraphPad Prism 9 (Graph Pad Software Co., CA, USA) was used for all statistical analyses. Normality of the data distribution was verified using the Shapiro-Wilk test. For the locomotion experiments, the effects of the different agonists were compared to vehicle injections by measuring the area-under-curve (AUC, from 30 to 150 min post ip injection; or from 15 to 135 min post brain injection) and analysed using Student’s t-tests or one-way ANOVA. For the other experiments, comparisons between groups were analysed using one-way ANOVA for repeated measures (5-HT_1A_ agonist testing) or two-way ANOVA for repeated measures (CNO treatment vs DREADD construct), using Geisser-Greenhouse correction for sphericity, followed, when necessary, by a Bonferroni multiple comparisons post-hoc test. A *p* value < 0.05 was considered significant.

## Results

### Systemic stimulation of Raphe nuclei 5-HT_1A_ autoreceptors reduces ethanol consumption

**Fig. 1:**
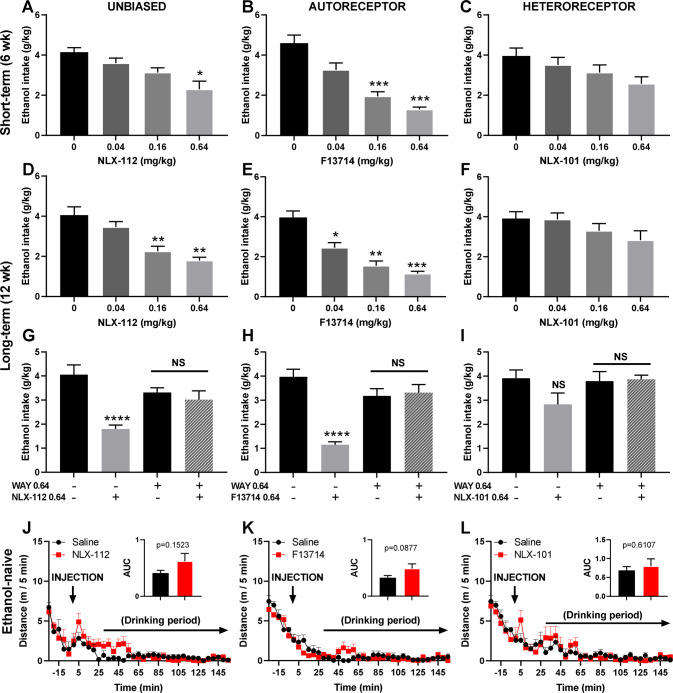
Short- and long-term ethanol intake is controlled by 5-HT_1A_ autoreceptors. **A–C** Short-term ethanol intake (6 weeks) is reduced by 5-HT1A receptor stimulation, with the highest dose of the unbiased agonist NLX-112 (**A**, repeated-measure one-way ANOVA, *n* = 8, F (1.760, 12.32) = 9.049, *p* = 0.0047, with Bonferroni multiple comparison: *: *p* = 0.0114 vs vehicle). This effect is likely mediated by the activation of 5-HT1A autoreceptors as ethanol intake is dose-dependently reduced by the autoreceptor agonist F13714 (**B**, repeated-measure one-way ANOVA, *n* = 8, F(1.585, 11.10) = 20.17, *p* = 0.0003, with Bonferroni multiple comparison: ***: *p* = 0.0006 (0.16 mg/kg) and *p* = 0.0003 (0.64 mg/kg) vs vehicle), but not by the heteroreceptor agonist NLX-101 (**C**, repeated-measure one-way ANOVA, *n* = 8, F(2.046, 14.32) = 2.446, *p* = 0.1211). D-I. Long-term ethanol intake (12 weeks) is reduced by 5-HT1A receptor stimulation, with a dose-dependent effect of the unbiased agonist NLX-112 (**D**, repeated-measure one-way ANOVA, *n* = 8, F(1.945, 13.61) = 12.03, *p* = 0.001, with Bonferroni multiple comparison: **p = 0.0028 (0.16 mg/kg) and *p* = 0.0038 (0.64 mg/kg) vs vehicle). Again, this effect is likely mediated by the activation of 5-HT1A autoreceptors as ethanol intake is dose-dependently reduced by the autoreceptor agonist F13714 (**E**, repeated-measure one-way ANOVA, *n* = 7, F(2.134, 12.80) = 24.27, *p* < 0.0001, with Bonferroni multiple comparison: *p = 0.044, **p = 0.0051, ***p = 0.0007), but not the heteroreceptor agonist NLX-101 (**F**, repeated-measure one-way ANOVA, *n* = 8 F(1.643, 11.50) = 2.252, *p* = 0.15). The specific involvement of 5-HT1A receptors has been confirmed by the blockade of the effects of the highest dose of NLX-112 (**G**, one-way ANOVA, *n* = 8, F(3, 28) = 9.622, *p* = 0.0002, with Bonferroni multiple comparison: ****p < 0.0001 vs veh/veh control), or F13714 (**H**, one-way ANOVA, *n* = 7, F(3, 24) = 18.33, *p* < 0.0001, with Bonferroni multiple comparison: ****p < 0.0001 vs veh/veh control) by the selective 5-HT1A receptor antagonist WAY100635 (0.64 mg/kg). The lack of efficacy of the highest dose of NLX-101 on ethanol intake showed also no effect of the WAY100635 (*n* = 8, **I**). **J**–**L** Effects of NLX-112, F13714 and NLX-101 on locomotor activity was assessed in ethanol-naïve mice, for 2.5 h after injection, or 2 h min into the drinking session, showing no effect on locomotor activity of NLX-112 (**J**, *t* test on the area-under-curve (AUC), *n* = 6, *p* = 0.1523), F13714 (**K**, *t* test on the area-under-curve, *n* = 6, *p* = 0.0877) or NLX-101 (**L**, *t* test on the area-under-curve, *n* = 6, *p* = 0.6107).

We and others have previously shown that the stimulation of 5-HT_1A_ receptors by the agonists pindolol, buspirone or tandospirone reduces ethanol intake in mice, rats and monkeys; [27, 28, 31, 48, 49] however, due to the complex pharmacology of these drugs, with lack of selectivity and specificity at auto- vs heteroreceptors, the respective contribution of these receptor subpopulations in ethanol consumption could not be determined. Therefore, we used highly selective unbiased (NLX-112) or biased agonists targeting 5-HT_1A_ autoreceptors (F13714) or heteroreceptors (NLX-101) to determine the respective involvement of these 5-HT_1A_ receptor subpopulations in ethanol consumption in mice following short- (6 weeks) or long-term (12 weeks) exposure to ethanol in the “drinking-in-the dark” model. We found that administered systemically (i.p. route), the highly selective (unbiased) 5-HT_1A_ receptor agonist NLX-112 reduces ethanol intake following short- (Fig. 1A) and long-term (Fig. 1D) exposure, as previously reported for the 5-HT_1A_ partial agonist tandospirone [28]. We further confirmed previous observations [27] that NLX-112 showed higher potency for long- rather than short-term ethanol exposure, with a minimum effective dose (MED) of 0.16 mg/kg vs 0.64 mg/kg for long-vs short-term ethanol exposure, respectively (Fig. 1A, D). Interestingly, we found that this effect was preferentially mediated by the stimulation of 5-HT_1A_ autoreceptors following both short- (Fig. 1B) and long-term (Fig. 1E) exposure to ethanol, with the autoreceptor-targeting agonist F13714 reducing short-term ethanol intake from 0.16 mg/kg (Fig. 1B), and long-term ethanol intake from 0.04 mg/kg (Fig. 1E). By contrast, the heteroreceptor-targeting agonist, NLX-101, did not alter ethanol intake following short- (Fig. 1C) nor long-term exposure (Fig. 1F), whatever the dose tested. Note that the absence of effects of NLX-101 is unlikely to be due to pharmacological underdosing, since NLX-101 is active at 0.16 or 0.64 mg/kg i.p. on sucrose intake in mice [42]. The specific contribution of 5-HT_1A_ receptors in these effects was confirmed by blocking the ethanol intake-reducing effects of the highest dose (0.64 mg/kg) of NLX-112 and F13714 on long-term ethanol intake with the selective 5-HT_1A_ receptor antagonist WAY 100,635 (0.64 mg/kg, Fig. 1G–I). The reducing effects of NLX-112 or F13714 on ethanol intake was most likely specific, since food (chow) or water consumption in food or water deprived naïve mice was unaffected at 0.64 mg/kg (supplementary Figure S1A-C). Similarly, there was no alteration in locomotor behaviour across the two-hour drinking period (Fig. 1J–L). Together these data point to somatodendritic 5-HT_1A_ autoreceptors as playing a major role in the control of ethanol intake.

### Dorsal and median Raphe nuclei 5-HT_1A_ autoreceptors mediate ethanol consumption following short- and long-term exposure, respectively

**Fig. 2:**
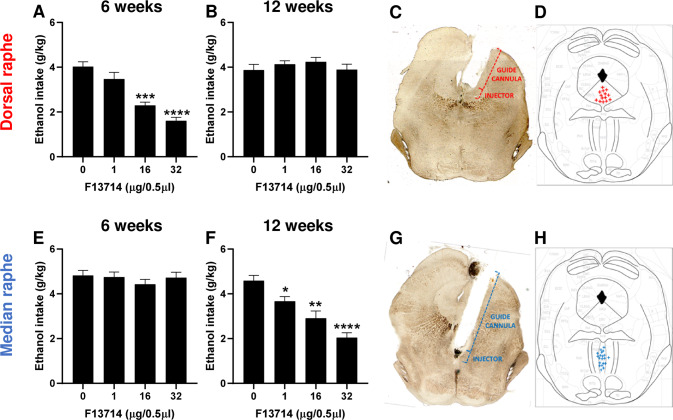
5-HT_1A_ autoreceptors in the dorsal and median raphe mediate short- and long-term ethanol intake, respectively. **A–D** Local infusion of F13714 in the dorsal Raphe nucleus (DRN) dose-dependently reduces ethanol intake following short-term (6 weeks) exposure (**A**, repeated measure one-way ANOVA, *n* = 14, F(2.054, 26.70) = 34.43, *p* < 0.0001, with Bonferroni multiple comparison: ***p < 0.001 and ****p < 0.0001 vs vehicle) but has no effects following long-term (12 weeks exposure, (**B**) repeated measure one-way ANOVA, *n* = 14, F(2.847, 37.01) = 1.255, *p* = 0.3035). Cannula placement was verified by histology (**C**) and only animals with DRN-targeting cannulae were included (**D**). Local infusion of F13714 in the median Raphe nucleus (MRN) has no effect on ethanol intake following short-term (6 weeks) exposure (**E**, repeated measure one-way ANOVA, *n* = 20, F(2.784, 52.89) = 1.277, *p* = 0.2915) but dose-dependently- reduces ethanol intake following long-term (12 weeks) exposure (**F**, repeated measure one-way ANOVA, *n* = 20, F(2.465, 46.83) = 20.13, *p* < 0.0001, with Bonferroni multiple comparison: *p = 0.0376, **p = 0.0026 and ****p < 0.0001 vs vehicle). Cannula placement was verified by histology (**G**) and only animals with MRN-targeting cannulae were included (**H**).

As 5-HT_1A_ autoreceptors are found both in the DRN and MRN, where they have differential sensitivity to agonist-induced inhibition of 5-HT neuronal activity [50], we investigated the respective contribution of DRN and MRN 5-HT_1A_ autoreceptors in ethanol intake following short- and long-term exposure, by delivering locally the autoreceptor agonist F13714 (0, 1, 16, 32 μg/0.5 μl) into either nucleus. We found that intra-DRN microinjection of F13714 dose-dependently reduced ethanol intake following short-term (6 weeks) exposure with a significant reduction at 16 and 32 μg (Fig. 2A). However, there was no effect of intra-DRN microinjection of F13714 on ethanol intake following long-term (12 weeks) exposure (Fig. 2B). Placements of microinjection cannulae were verified by histology (Fig. 2C) and only animals with correct placements were included in the analysis (Fig. 2D). As opposed to intra-DRN microinjection, we found that intra-MRN injection of F13714 had no effect on ethanol intake following short-term exposure (Fig. 2E), but dose-dependently reduced ethanol intake following long-term exposure with significant effects of all the doses (Fig. 2F). Placement of the microinjection cannulae were verified by histology (Fig. 2G) and only animals with correct placements were included in the analysis (Fig. 2H). The reducing effects of DRN and MRN microinjections of F13714 on ethanol intake was most likely specific, since food (chow) consumption in food-deprived naïve mice was unaffected by the highest dose (32 µg, supplementary Figure S1D-F). These results indicate that a switch takes place from DRN to MRN and that subpopulations of 5-HT_1A_ autoreceptors are differentially involved in ethanol consumption between short- and long-term ethanol exposure.

### Chemogenetic inhibition of dorsal and median Raphe nuclei 5-HT neuronal activity differentially reduces short- and long-term ethanol consumption, respectively

**Fig. 3:**
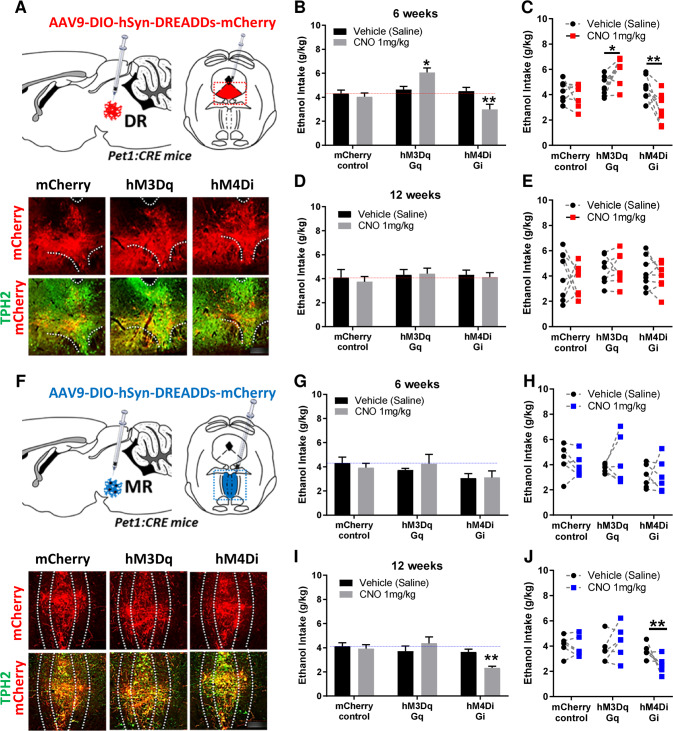
Chemogenetic modulations of dorsal and median Raphe 5-HT neuron activity differentially affect short and long-term ethanol consumption. **A**–**E** mCherry-control, hM3Dq-excitatory and hM4Di-inhibitory DREADDs were injected in *pet1*-5-HT DRN neurons and their expression in TPH2-immunoreactive neurons was confirmed by immunohistochemistry (**A**, micrograph field corresponding to the red dashed square in the diagram above, scale bar: 150 µm). Manipulation of DREADD-expressing neurons in the DRN by systemic CNO (1 mg/kg) bidirectionally modulated short-term (6 weeks) ethanol intake, with their stimulation increasing ethanol intake, and their inhibition reducing ethanol intake (**B**–**C**, repeated measure two-way ANOVA, *n* = 8, treatment x construct: F (2, 21) = 11.34; *p* = 0.0005; with Bonferroni multiple comparison: *p = 0.0113, **p = 0.0072). However, this modulation of ethanol intake by DRN 5-HT neurons was lost long-term exposure to ethanol (**D**–**E**, repeated measure two-way ANOVA, *n* = 8, treatment x construct: F (2, 21) = 0.1371; *p* = 0.8727). mCherry-control, hM3Dq-excitatory and hM4Di-inhibitory DREADDs were then injected in *pet1*-5-HT MRN neurons and their expression in TPH2-immunoreactive neurons was confirmed by immunohistochemistry (**F**, micrograph field corresponding to the blue dashed square in the diagram above, scale bar: 100 µm). Manipulation of DREADD-expressing neurons in the MR by intraperitoneal CNO (1 mg/kg) had no effect on short-term (6 weeks) ethanol intake (**G**–**H**, repeated measure two-way ANOVA, *n* = 6, treatment x construct: F (2, 15) = 0.5862, *p* = 0.5687). However, inhibition of MRN 5-HT neurons by systemic CNO (1 mg/kg) reduced long-term ethanol intake (**I**–**J**, repeated measure two-way ANOVA, *n* = 6, treatment x construct: F (2, 15) = 9.318, *p* = 0.0023, with Bonferroni multiple comparison: **p = 0.0031), while their activation had no effect (**I-J**, *p* = 0.1836).

To investigate whether modulating 5-HT neuron activity in the DRN or the MRN could affect short- and long-term ethanol drinking, we used a chemogenetic approach, the Designer Receptors Exclusively Activated by Designer Drugs (DREADDs). For this, we expressed three different CRE-dependent DREADDs constructs using AAV9 serotype particles (DIO-hSyn-mCherry, DIO-hSyn-**hM3Dq**-mCherry and DIO-hSyn-**hM4Di**-mCherry) as control, excitatory and inhibitory DREADDs, respectively, in *pet1* 5-HT neurons (*pet1*-CRE mice). To control for any off-target effects of the designer drug Clozapine-N-Oxide (CNO), mice that received mCherry control constructs were also treated with the same dose of CNO (1 mg/kg i.p.). Following DRN delivery of AAV9 particles, we verified the correct expression of the 3 DREADDs constructs in TPH2 immunoreactive 5-HT neurons of the DRN (Fig. 3A, Supplementary Fig. S2). Upon stimulation of the DREADDs constructs by CNO in mice exposed to ethanol for 6 weeks, we observed a significant treatment x construct interaction effect on ethanol intake. Post-hoc multiple comparison tests revealed a significant increase in ethanol intake following stimulation of DRN 5-HT neurons and a significant decrease in ethanol intake following silencing of DRN 5-HT neurons (Fig. 3B). There was no effect of CNO in mCherry control mice when compared to mCherry control mice treated with saline. Interestingly, there was no effect of manipulating DRN 5-HT neuronal activity in the same animals that continued to consume ethanol for a total of 12 weeks (treatment x construct interaction effect) (Fig. 3D). Next, following MRN delivery of AAV9 particles we verified the correct expression of the 3 DREADDs constructs in TPH2 immunoreactive 5-HT neurons of the MRN (Fig. 3F, Supplementary Fig. S3). Upon stimulation of the DREADDs constructs by CNO (1 mg/kg) in mice exposed to ethanol for 6 weeks, there was no effect of manipulating MRN 5-HT neuron activity on ethanol consumption (treatment x construct) (Fig. 3G). However, we observed a significant treatment x construct interaction effect on ethanol intake when MRN neuron activity was modulated in long-term ethanol consuming mice (12 weeks) (Fig. 3I). Multiple comparison post-hoc tests revealed a significant decrease in ethanol intake following silencing of MR 5-HT neurons. The effects of chemogenetic modulation of 5-HT neuron activity are likely specific to ethanol consumption as stimulating or inhibiting DRN (6 weeks) or MRN (12 weeks) had no effects on sucrose consumption (supplementary Figure S1G-J). These results provide further evidence that 5-HT neuron activity in the DRN plays a role in short-term ethanol intake, and that it switches to MRN following long-term ethanol intake.

### Long-term consumption of ethanol alters 5-HT innervation in the DG in a 5-HT_1A_ receptor-dependent manner

**Fig. 4:**
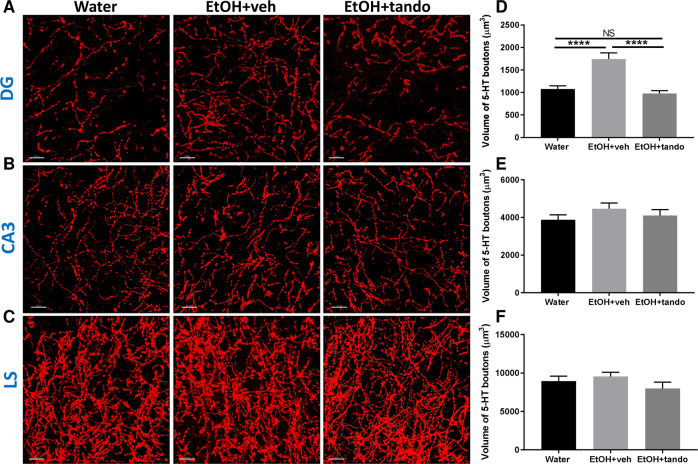
Long-term ethanol exposure alters 5-HT neurons innervation in the DG and is reversed by chronic 5-HT_1A_ agonist treatment. 5-HT-immunoreactive axons (red) from the dentate gyrus (DG; **A**), the CA3 region of the hippocampus (CA3; **B**) and lateral septum (LS; **C**) of mice exposed to water (water; *left panel*), ethanol—treated with vehicle (EtOH + veh, *middle panel)* or ethanol—chronically treated with the 5-HT_1A_ agonist tandospirone (EtOH + tando; right panel) were labelled and the varicosities reconstructed in 3D. Scale bar: 15 μm. Total volume of reconstructed varicosities of mice exposed to water (water; *black*), ethanol - treated with vehicle (EtOH + veh, light grey*)* or ethanol - chronically treated with the 5-HT1A agonist tandospirone (EtOH + tando; dark grey) was quantified in the DG (**D**), CA3 (**E**) and LS **(F**). Mean volume ± SEM (in μm^3^). One-way ANOVA, *n* = 6, ****p < 0.0001, NS non-significant, *p* > 0.9999.

We observed above that 5-HT_1A_ autoreceptors from the MRN control ethanol intake under long-term exposure conditions. The 5-HT innervation ascending from the MRN projects densely to the dentate gyrus (DG), the CA3 region of the hippocampus and the lateral septum (LS) [51–53]. We therefore investigated the effect of long-term ethanol consumption on 5-HT innervation of these projection brain regions. Following 12 weeks of ethanol consumption, 5-HT-immunoreactive axons were labelled, 3D-reconstructed (Fig. 4A–C) and quantified (Fig. 4D–F) as previously described [46, 47, 54, 55]. The volume of 5-HT varicosities (boutons) in the DG, CA3 and LS was quantified in water-exposed animals (Water), ethanol-exposed animals chronically treated (2 weeks, following 12 weeks of consumption) with either vehicle (EtOH + veh) or with the 5-HT_1A_ receptor partial agonist, tandospirone at 3 mg/kg/day, i.p. (EtOH + tando) (Fig. 4D–F). We found that long-term ethanol consumption significantly increases the volume of 5-HT-immunoreactive boutons in the DG (Fig. 4D) but not in the CA3 (Fig. 4E) or LS (Fig. 4F). Interestingly chronic treatment with the 5-HT_1A_ agonist tandospirone prevented this increase in the DG and restored the volume of 5-HT varicosities to the level of water-control animals (Fig. 4D) while having no effect on 5-HT innervation in the CA3 or LS. This suggests that long-term consumption of ethanol produces activity-dependent alterations in the morphology of 5-HT^MRN>DG^ neurons that likely contribute to the maintenance of long-term ethanol intake.

### 5-HT^MRN→DG^ neuronal circuit activity modulates long-term ethanol intake

**Fig. 5:**
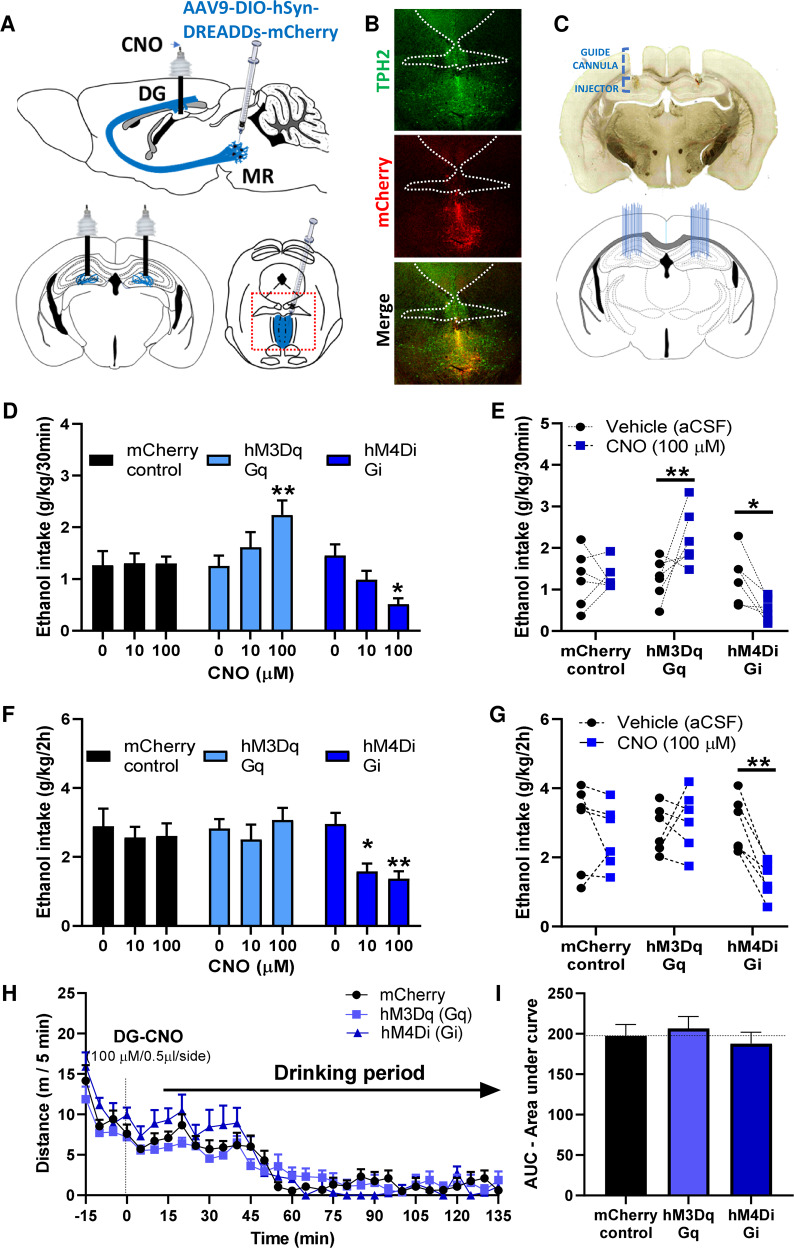
Chemogenetic manipulation of 5-HT^MRN>DG^ circuit modulates ethanol intake following long-term exposure. **A–C** mCherry-control, hM3Dq-excitatory, and hM4Di-inhibitory DREADDs were injected in *pet1*-5-HT MRN neurons and bilateral cannulae were implanted in the hippocampus, above the dentate gyrus (**A**). Expression of DREADD constructs in TPH2-immunoreactive MRN neurons was verified by immunohistochemistry (**B**, field corresponding to the red dashed square in A) and the correct cannulae placement verified by histology (**C**). Chemogenetic manipulation of 5-HT^MRN>DG^ neuron terminals by local infusion of CNO (100 μM) bidirectionally modulated the binge-consumption of ethanol (first 30 min of a 2 h drinking period) (**D–E**, repeated measure two-way ANOVA, *n* = 6, treatment x construct: F(4, 30) = 6.545, *p* = 0.0007). Bonferroni multiple comparison showed that the stimulation of these terminals increased the 30 min intake of ethanol (**D–E**, **p = 0.074), while their inhibition reduced the binge-intake of ethanol (**D–E**, *p = 0.013). Chemogenetic manipulation of 5-HT^MRN>DG^ neuron terminals also modulated the 2 h consumption of ethanol (**F–G**, repeated measure two-way ANOVA, *n* = 6, treatment: F(1.883, 28.25) = 3.902, *p* = 0.0341), with Bonferroni multiple comparison showing that only the inhibition of these terminals reduced the overall 2 h intake of ethanol (**F–G** *p = 0.0239; **p = 0.0076), but no effect of chemogenetic activation (*p* > 0.99). Effect of intra-DG injection of CNO on locomotor activity (**H**) showing no effect over the 2-hour drinking period (**I**, One-way ANOVA on AUC [15–135 min], *n* = 4, F (2, 9) = 0.4343, *p* = 0.6606).

To determine the role played by 5-HT^MRN>DG^ neurons in the maintenance of long-term ethanol drinking behaviour, we used chemogenetics (AAV-DIOhSyn-DREADDs-mCherry constructs) in the MRN, combined with local microinjections of the designer drug CNO in the DG to investigate the effect of specific stimulation (hM3Dq construct variant) or silencing (hM4Di construct variant) of the activity of this neuronal circuit on ethanol intake following long-term exposure (Fig. 5A). The correct expression of the viral construct in the MRN was verified by immunohistochemistry (Fig. 5B) and the correct placements of guide-cannulae in the DG were verified by histology (Fig. 5C). We observed that the stimulation of 5-HT^MRN→DG^ neurons by the highest concentration of CNO (100 μM or 20 ng/0.5 µl) significantly increases, while silencing of 5-HT^MRN→DG^ neurons significantly decreases ethanol intake, during the initial binge-phase of ethanol drinking sessions (first 30 min of the 2 h session) (Fig. 5D–E). The initial increase in ethanol intake elicited by 5-HT^MRN→DG^ neurons activation by CNO (100 μM) did not persist over the entire drinking session whereas inhibition of 5-HT^MRN→DG^ neurons dose-dependently reduced ethanol intake for the whole 2 h (Fig. 5F–G). We then assessed whether these alterations in drinking behaviour were mediated by changes in locomotor activity after DG injection of CNO (100 μM). We found that the stimulation of 5-HT^MRN>DG^ neurons by hM3Dq-CNO or silencing by hM4Di-CNO did not affect overall locomotor activity compared to mCherry-control mice (Fig. 5H–I). This suggests that the reduction in ethanol intake is not resulting from altered locomotor behaviour.

## Discussion

The present study reveals that long-term ethanol intake changes the role of 5-HT_1A_-autoreceptor-dependent activity in the DRN and the MRN. As the length of ethanol exposure increases, there is a switch from a DRN- to MRN-mediated control of ethanol intake. This switch is likely mediated by ethanol intake-induced changes in 5-HT innervation observed in dentate gyrus (DG) of the hippocampus, as we have shown that 5-HT^MRN→DG^ circuits mediate long-term ethanol consumption.

Our laboratory has previously demonstrated the 5-HT_1A_ receptor-dependent modulation of ethanol consumption, without determining the respective involvement of 5-HT_1A_ auto- or heteroreceptors in this mechanism [27, 28, 31, 56]. The present study showed for the first time the exclusive contribution of 5-HT_1A_ autoreceptors in ethanol intake. Systemic treatments with the 5-HT_1A_ autoreceptor agonist F13714 showed increased efficacy to reduce ethanol intake between short- and long-term ethanol consumption, suggesting a change in 5-HT_1A_ autoreceptor function after long-term ethanol consumption. We further demonstrated a switch in the role of DRN to MRN 5-HT over time, with DRN-5-HT_1A_ autoreceptor stimulation reducing short-term ethanol intake drinking, and MRN-5-HT_1A_ autoreceptor stimulation reducing long-term ethanol intake. This suggests that the two Raphe nuclei are differentially involved in ethanol intake following short- and long-term.

5-HT_1A_ autoreceptors play an important role in the regulation of the activity of 5-HT neurons located in either the DRN and MRN [57], where they mediate various physiological function such as reward processing, anxiety-, stress-, or fear-related behaviour [20, 58–63]. Using local microinjections of the selective 5-HT_1A_ autoreceptor biased agonist F13714, we observed a functional transition in the role of DRN to MRN in the maintenance of short- and long-term alcohol consumption, with DRN-5-HT_1A_ autoreceptor stimulation only reducing short-term drinking, while long-term drinking was reduced by MRN-5-HT_1A_ autoreceptor stimulation. This suggests that the two Raphe nuclei are differentially involved in alcohol drinking behaviour following short- and long-term exposure. This time-dependent recruitment of DRN and MRN 5-HT_1A_ autoreceptors could be due to differences in their propensity to hypersensitization following acute or chronic alcohol exposure [64], or in their variable responses to agonist stimulation [64, 65]. Interestingly, a similar shift in 5-HT_1A_ receptor function has been observed, also from DRN to MRN, in the psychostimulant responses to chronic cocaine [66]. In addition, while nicotine-withdrawal following short-term exposure (7 days) recruits DRN 5-HT neurons [67], it is likely that nicotine-withdrawal following longer exposure (6 weeks) is mostly controlled by MRN 5-HT neuron activity [68], suggesting that a neuroadaptive shift between DRN and MRN neurotransmission could mediate the transitioning from short-term consumption to long-term substance abuse. However, an opposite shift, from MRN to DRN, has also been demonstrated in the transition from moderate to compulsive cocaine intake following SERT selective knock down in the different raphe nuclei [69]. Therefore, whether the functional shift between the DRN to MRN could underly a specific mechanism by which moderate intake of ethanol evolves into chronic binge-like or compulsive consumption remains to be elucidated.

However, raphe nuclei also contain non-serotonergic cells that express the 5-HT_1A_ receptors, such as GABA interneurons in the DRN [70] or other undefined cell types in the MRN [71]. To determine the specific involvement of serotonergic neurons, we further investigated the functional DRN to MRN switch using chemogenetics, confirming that silencing of DRN-5-HT neurons reduces ethanol intake in short-term but not long-term alcohol-exposed mice. Conversely, silencing of MRN-5-HT neurons does not affect short-term alcohol intake while it does reduce long-term consumption. 5-HT_1A_ autoreceptor stimulation is inhibitory of 5-HT neuron activity [72], therefore, the chemogenetic data corroborates our results with the 5-HT_1A_ autoreceptor biased agonist F13714. Moreover, these observations suggest that alcohol consumption elicits hyperactivity of 5-HT neurons in the Raphe nuclei, as previously reported [30], first initiated in the DRN following short-term consumption, and then transferred to MRN after long-term alcohol consumption.

Raphe 5-HT_1A_ autoreceptor sensitization or upregulation has been observed in mice, rats and monkeys following chronic alcohol consumption [26, 73, 74]. In line with an increased 5-HT_1A_ autoreceptor inhibitory function, microdialysis studies have shown that while acute/short-term ethanol intake elevates 5-HT release in various brain regions, including the hippocampus, nucleus accumbens, striatum, amygdala, prefrontal cortex, and ventral tegmental area, chronic/long-term exposure to ethanol produces a reduction of extracellular 5-HT levels and/or 5-HT turnover in these brain regions (for review see [25]). This suggests that long-term ethanol consumption is linked to an overall reduction in 5-HT neurotransmission, however, people abusing alcohol likely have an increased expression of the tryptophan hydroxylase 2 (TPH2, the rate-limiting biosynthetic enzyme for 5-HT) [75, 76], reduced expression of the monoamine oxidase A (MAO-A, the catalysis enzyme for 5-HT) [77], and increased rates of 5-HT neuronal uptake [78, 79], which rather suggests increased 5-HT levels, presumably intracellularly. For instance, inhibitors of MAO activity have been shown to increase the intracellular content of 5-HT within serotonergic axons, in the cortex, hippocampus and thalamus [80]. Our results showing increased 5-HT immunoreactive innervation within the dentate gyrus (DG) of the hippocampus support an elevation of intracellular 5-HT levels and therefore suggest that long-term ethanol consumption augments the levels of axonal 5-HT while likely reducing its extracellular levels.

Previous work from our laboratory has demonstrated that long-term ethanol consumption elicits 5-HT_1A_ receptor-dependent deficits in anxiety-like behaviour and hippocampal neurogenesis in the DG [28], a hallmark of chronic alcohol abuse [81]. Although serotonergic drugs that ameliorate neurogenesis have shown limited clinical efficacy in the treatment of alcohol dependence, we now confirm by a local chemogenetics approach, that the 5-HT^MRN→DG^ neuronal circuit is involved in long-term ethanol consumption, showing that its inhibition reduces ethanol intake under these conditions. There is increasing evidence indicating that the hippocampus contributes to drug-reward processes, drug-related memory formation, and drug-induced anxiety and dysphoria (for review see [82]). It is likely that neuroadaptations produced by prolonged substance abuse augment hippocampal activity, thus amplifying the responses to substances of abuse and associated cues [82]. Hence, long-term serotonergic neuroadaptations within the hippocampus, and possibly the resulting changes in neurogenesis, may contribute to relapse vulnerability [83] through enhanced drug sensitivity, enhanced drug memory, or anxiogenic stimuli. It is noteworthy that one limitation of the present study is the use of male mice only. Therefore, since alcohol drinking behaviour, anxiety, as well as the regulation of 5-HT signalling, have shown important sex differences, further work is needed to provide a detailed characterization of the gender-specific role played by DRN and MRN 5-HT neurotransmission in alcohol binge consummatory behaviour.

## Conclusion

Using a dual pharmacological and chemogenetic approach, the present study reveals that DRN and MRN 5-HT neurons are differentially involved in alcohol drinking behaviour whether it results from short- or long-term exposure, and that the functional transition from DRN to MRN-mediated behaviour might represent a mechanism by which acute alcohol consumption develops into chronic binge-like drinking behaviour. Our chemogenetic data being in complete adequation with the pharmacological data, the present study further indicates that targeting Raphe nuclei 5-HT_1A_ autoreceptors with selective and potent agonists might represent an innovative pharmacotherapeutic strategy to combat alcohol abuse. Although the 5-HT_1A_ autoreceptor preferential agonist F13714 showed a higher potency in reducing ethanol intake, this compound cannot be developed clinically. However, the NLX-112 compound, which reduces both short- and long-term alcohol consumption is clinically ready to be tested as a promising treatment for alcohol use disorders. Moreover, the biopharmaceutical company, Neurolixis, is currently conducting a drug discovery program on 5-HT_1A_ receptor biased agonists, with some new chemical entities showing various profiles of biased agonism. Further work with these new compounds may unravel even more efficacious therapeutic strategies for AUDs.

## Supplementary information

SUPPLEMENTARY FIGURE LEGENDS

SUPPLEMENTARY METHOD

SUPP FIG S1 :

SUPP FIG S2 :

SUPP FIG S3 :
