# Supplementary material for: Neural serotonergic circuits for controlling long-term voluntary alcohol consumption in mice
Source: Mol Psychiatry. 2022 Oct 4;27(11):4599–610. doi: 10.1038/s41380-022-01789-z (PMC9531213; doi:10.1038/s41380-022-01789-z)
Supplement: Supplementary file 1 — SUPPLEMENTARY FIGURE LEGENDS [file 41380_2022_1789_MOESM1_ESM.docx]

**SUPPLEMENTARY FIGURE LEGENDS**

**SUPPLEMENTARY FIGURE S1:** Systemic injections of NLX-112 and F1371 (0.64 mg/kg, i.p.) do not alter appetitive behavior, as shown by the absence of effect on chow intake (**A**, one-way ANOVA, n=5, F(2, 12)=0.3896, p=0.6856), water intake (**B**, one-way ANOVA, n=5, F(2, 12)=0.3628, p=0.7031) and 25% sucrose intake in sucrose-naïve animals (**C**, one-way ANOVA, n=5, F(2, 12)=0.5153, p=0.6100). Appetitive behavior is not altered by brain microinjections of F13714 (32 µg/0.5µl) in the DRN (**D**, paired two-tailed Student t-test, n= 4, p=0.6997) or the MRN (**E**, paired two-tailed Student t-test, n= 4, p=0.9144). Correct cannula placements were confirmed in the DRN and MRN of all animals by histology (**F**). Chemogenetic manipulation of DRN (G-H) 5-HT neurons by hM3Dq (Gq) or hM4Di (Gi) following systemic injection of CNO (1 or 5 mg/kg, i.p.) did not alter 30 min (**G,** two-way ANOVA in repeated measures, n=6, treatment x genotype: F (6, 40) = 0.5634, p=0.7567) or 2 h (**H,** two-way ANOVA in repeated measures, n=6, treatment x genotype: F (6, 40) = 0.4931, p=0.8096) intake of 25% sucrose following 6 weeks of exposure. Chemogenetic manipulation of MRN (**I-J**) 5-HT neurons by hM3Dq (Gq) or hM4Di (Gi) following systemic injection of CNO (1 or 5 mg/kg, i.p.) did not alter 30 min (**I,** two-way ANOVA in repeated measures, n=6, treatment x genotype: F (6, 40) = 0.2493, p=0.9567) or 2 h (**J,** two-way ANOVA in repeated measures, n=6, treatment x genotype: F (6, 40) = 0.2239, p=0.9667) intake of 25% sucrose following 12 weeks of exposure.

**SUPPLEMENTARY FIGURE S2:** Representative micrographs showing the spread of the AAV9-mCherry reporter expression (**A-C**) around the DRN, showing a restricted reporter expression (**A**, mCherry, red) in TPH2-positive neurons (**B**, TPH2. green) in the DRN at lower magnification (scale bar= 500 µm). Representative micrographs corresponding to the white dashed box in A, B C, showing the cellular localization of the AAV9-mCherry reporter expression (**A-C**) in the DRN, showing the colocalization of the reporter expression (**A**, mCherry, red) in TPH2-positive cell bodies (**B**, TPH2. green) in the DRN at higher magnification (scale bar= 50 µm, aq=aqueduct).

**SUPPLEMENTARY FIGURE S3:** Representative micrographs showing the spread of the AAV9-mCherry reporter expression (**A-C**) around the MRN, showing a restricted reporter expression (**A**, mCherry, red) in TPH2-positive neurons (**B**, TPH2. green) in the MRN at lower magnification (scale bar=500 µm). Representative micrographs corresponding to the white dashed box in A, B C, showing the cellular localization of the AAV9-mCherry reporter expression (**A-C**) in the MRN, showing the colocalization of the reporter expression (**A**, mCherry, red) in TPH2-positive cell bodies (**B**, TPH2. green) in the MRN at higher magnification (scale bar= 50 µm, aq=aqueduct).
