# Supplementary material for: Neural serotonergic circuits for controlling long-term voluntary alcohol consumption in mice
Source: Mol Psychiatry. 2022 Oct 4;27(11):4599–610. doi: 10.1038/s41380-022-01789-z (PMC9531213; doi:10.1038/s41380-022-01789-z)
Supplement: Supplementary file 2 — SUPPLEMENTARY METHOD [file 41380_2022_1789_MOESM2_ESM.docx]

**SUPLEMENTARY METHOD**

Effects of systemic F13714 and NLX-112 (0.64 mg/kg, ip, 30 min before testing) and intra-raphe microinjection of F13714 (32 µg/0.5 µl, intra-DRN or intra-MRN, 15 min before testing) were assessed on appetitive behavior, i.e., food (chow) consumption and water intake, in ethanol naïve mice. Effects of systemic F13714 and NLX-112 (0.64 mg/kg, ip, 30 min before testing) was also assessed on 25% sucrose intake, in sucrose and ethanol naïve mice. Effects of chemogenetic modulation of DRN and MRN pet-1/5-HT neurons was also assessed on 25% sucrose intake, following 6 weeks (DRN) or 12 weeks (MRN) of exposure.

**Chow consumption:** Mice (n=5) were food deprived for 16 hours before systemic injections or intra Raphe nuclei microinjections. Following either systemic or intra-raphe microinjection, a pellet of chow was placed in the middle of the cage. Pellets were weighed prior and at the end of the 2 h session. Mice were weighed before the session. Chow consumption was measured as g of chow per kg of mouse body weight (g/kg).

**Water intake:** Mice (n=5) were water deprived for 10 h before systemic injections. Water was presented in 50 ml plastic falcon tubes (Corning Centristar, NY, USA) fitted with rubber stoppers and a 6.35 cm stainless-steel sipper tube with double ball bearings. Bottles were weighed prior to the presentation and after 2 h. Mice were weighed before the session. Water intake (in g) was converted in L of water per kg of mouse body weight (L/kg).

**Sucrose consumption:** Mice (n=5) were food and water deprived for 10 h before systemic injections. A solution of 25% sucrose (w/v) was presented in 50 ml plastic falcon tubes (Corning Centristar, NY, USA) fitted with rubber stoppers and a 6.35 cm stainless-steel sipper tube with double ball bearings. Bottles were weighed prior to the presentation and after 2 h. Mice were weighed before the session. Sucrose solution intake (in g) was converted in g of sucrose per kg of mouse body weight (g/kg).

**Sucrose consumption for the DREADDs experiments:** Stereotaxic surgeries were performed as described in the main Material and Method section, but instead of using the CNO-unresponsive AAV9-hSyn-DIO-mcherry constructs to control for CNO off-target effects, *pet-*CRE -/- mice (WT) were used as controls for AAV9-hSyn-DIO-hM3Dq-mcherry (Gq) and AAV9-hSyn-DIO-hM4Di-mcherry (Gi). Sucrose consumption was performed under the DID protocol as described in the main Material and Method section, except the mice were given a solution of 25% sucrose (w/v) for 6 or 12 weeks instead of ethanol, and CNO was tested at 1 and 5 mg/kg (ip).
