# Supplementary figures and images for: Neural serotonergic circuits for controlling long-term voluntary alcohol consumption in mice
Source: Mol Psychiatry. 2022 Oct 4;27(11):4599–610. doi: 10.1038/s41380-022-01789-z (PMC9531213; doi:10.1038/s41380-022-01789-z)

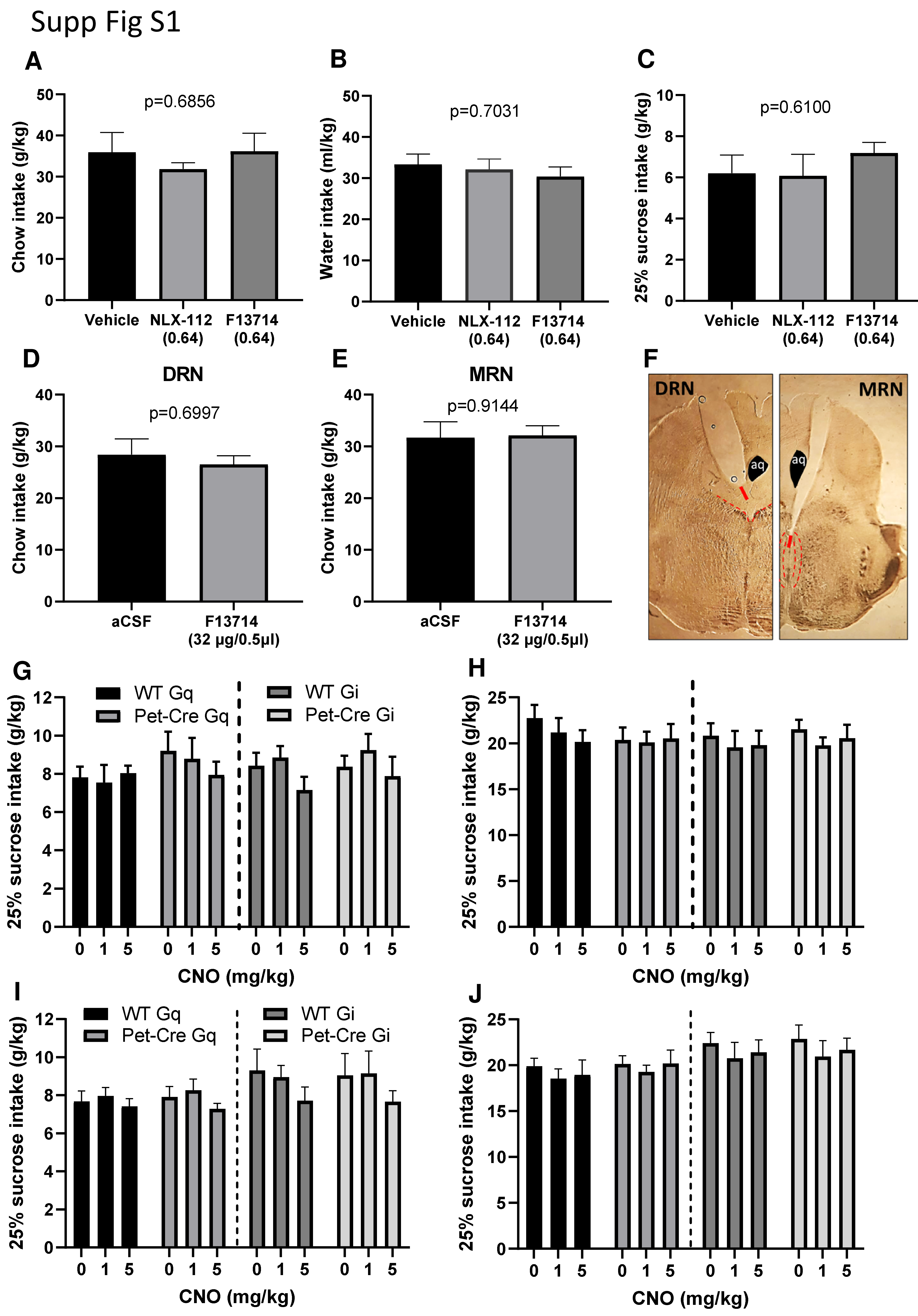

Supplement: Supplementary file 3 — SUPP FIG S1 : [file 41380_2022_1789_MOESM3_ESM.tif]

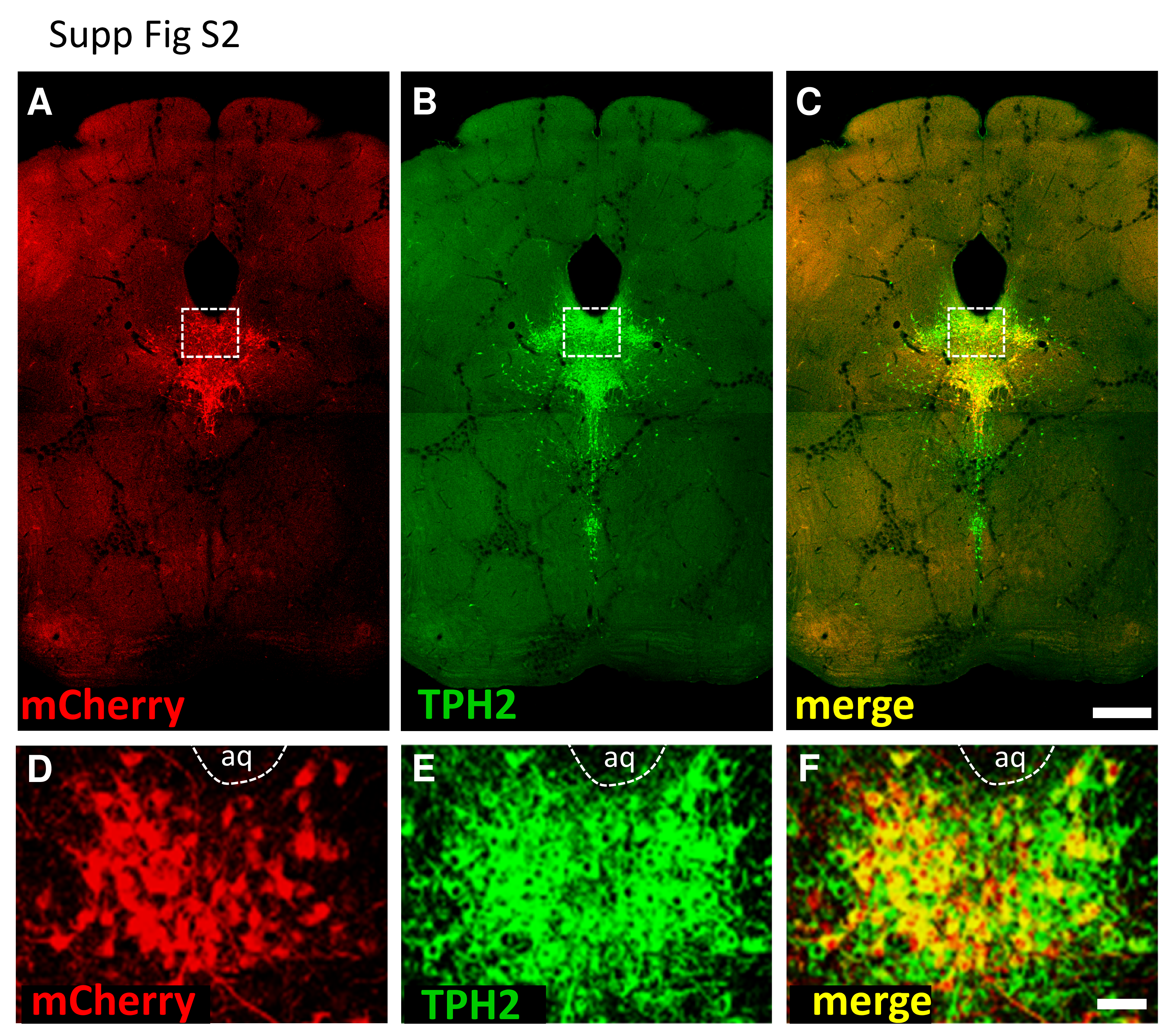

Supplement: Supplementary file 4 — SUPP FIG S2 : [file 41380_2022_1789_MOESM4_ESM.tif]

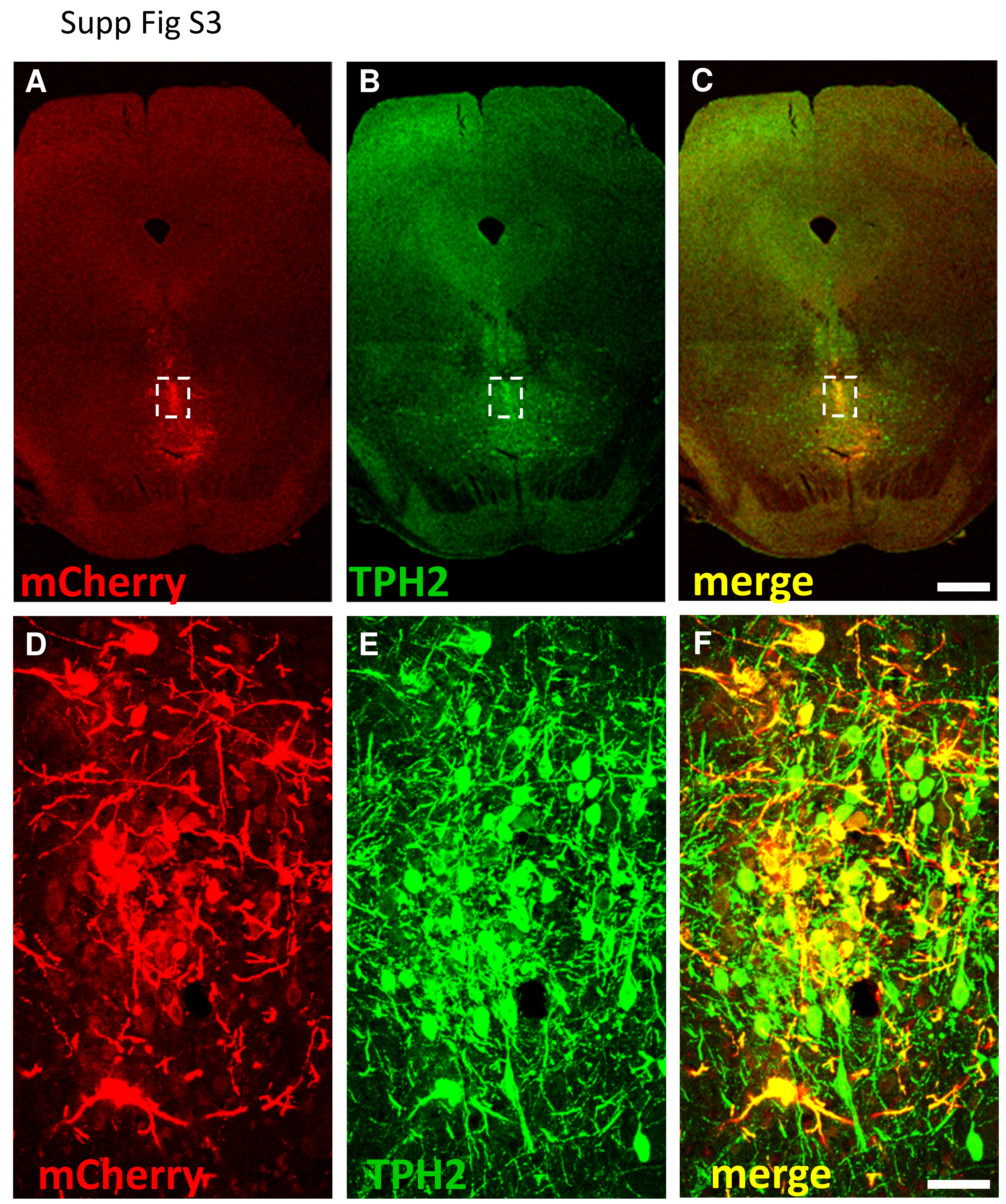

Supplement: Supplementary file 5 — SUPP FIG S3 : [file 41380_2022_1789_MOESM5_ESM.tif]
